# miR-144-3p Promotes Adipogenesis Through Releasing C/EBPα From Klf3 and CtBP2

**DOI:** 10.3389/fgene.2018.00677

**Published:** 2018-12-19

**Authors:** Linyuan Shen, Qiang Li, Jinyong Wang, Ye Zhao, Lili Niu, Lin Bai, Surong Shuai, Xuewei Li, Shunhua Zhang, Li Zhu

**Affiliations:** ^1^College of Animal Science and Technology, Sichuan Agricultural University, Chengdu, China; ^2^Sichuan Province General Station of Animal Husbandry, Chengdu, China; ^3^Chongqing Academy of Animal Science, Chongqing, China

**Keywords:** miR-144-3p, adipocytes, proliferation, differentiation, Klf3, CtBP2

## Abstract

MicroRNAs (miRNAs), a class of small non-coding RNAs, have been proved as novel and potent regulators of adipogenesis. A previous study has found out that miR-144-3p was a biomarker of type 2 diabetes, but the role of miR-144-3p in regulating adipogenesis was still unclear. In the present study, the expression of miR-144-3p increased in obese mice and during the 3T3-L1 differentiation process. Overexpression of miR-144-3p suppressed the expression of cell cycle regulatory factors and inhibited pre-adipocytes proliferation. Besides, overexpression of miR-144-3p accelerated lipid accumulation in adipocytes and positively regulated adipogenesis, which was also accompanied by increasing the expression of genes related to fatty acid synthesis and decreasing the expression of genes involved in fatty acid oxidation. Furthermore, luciferase activity assays indicated that miR-144-3p directly targeted Klf3 and CtBP2. The process was also confirmed by the mRNA and protein expression of Klf3 and CtBP2, which were suppressed by miR-144-3p. Furthermore, miR-144-3p targeting Klf3/CtBP2 would induce C/EBPα activity by releasing corepressors (Klf3 and CtBP2) from its promoter region. Moreover, we also observed that miR-144-3p could promote adipogenesis in mice injected with miR-144-3p agomir through tail-vein injection. Taken together, these results support that miR-144-3p can facilitate adipogenesis both *in vitro* and *in vivo*, which implies that miR-144-3p could be a target for therapeutic intervention in obesity and metabolic syndrome in the future.

## Introduction

Epidemic obesity is a threat to human health in both developed and developing countries (Zimmet et al., 2001), and it is also associated with increased risks of type II diabetes, hypertension, and cardiovascular disease (Kopelman, 2000; Goran et al., 2003). Adipocytes are a major cellular component in fat tissue. It is well known that during the development of obesity, there is an inflammatory reaction of the adipose tissue together with an impairment of adipose cells differentiation (McLaughlin et al., 2007; Arner et al., 2011; Cordido et al., 2014; Hammarstedt et al., 2018). Moreover, adipocytes are emerging as one of the major targets in the prevention and treatment of obesity and related metabolic syndrome (Grundy, 2004). To this end, it is important to understand the molecular genetic mechanisms that regulate adipogenesis. During the last decade, researchers have found out that adipogenesis was governed by a tightly controlled cascade of transcription factors, which coordinated hundreds of downstream genes (Farmer, 2006). In general, CCAAT/enhancer binding protein (C/EBP) β and C/EBPδ are first activated during the early stage of adipogenesis, which will directly activate C/EBPα and peroxisome proliferator-activated receptor γ (PPARγ) expression. Then, the latter factors, C/EBPα and PPARγ, will induce each other’s expression by a positive feedback loop, which results in activating downstream target genes related to promoting and maintaining adipogenesis [such as adipocyte fatty acid-binding protein 2 (aP2)] (Rosen et al., 2000; Rosen and MacDougald, 2006).

microRNAs (miRNAs), a class of non-coding endogenous RNA molecules, post-transcriptionally regulate gene expression by binding to 3′UTR of specific mRNA targets, which results in either a translational repression or a cleavage of the affected mRNA (Bartel, 2004). Interestingly, some miRNAs have been verified to affect adipocyte biology, adipocyte differentiation, and adipose tissue function. For instance, miR-143, miR-17-92 cluster, miR-30c, and miR-24 promote adipocyte differentiation by negatively regulating extracellular signal-regulated kinase 5 (*ERK5*) (Esau et al., 2004), tumor-suppressor *Rb2/p130* (Wang et al., 2008), activin receptor-like kinase 2 (*ALK2*) (Karbiener et al., 2011), and mitogen-activated protein kinase 7 (*MAPK7*) (Jin et al., 2016), respectively. miR-27b, miR-200b, miR-26b, and miR-215 impair adipocyte differentiation by targeting *PPARγ* (Karbiener et al., 2009), Kruppel like factor 4 (*Klf4*) (Shen et al., 2018), phosphatase, tensin homolog gene (*PTEN*) (Song et al., 2014), and fibronectin type III domain containing 3B (*FNDC3B*) (Peng et al., 2016), respectively.

A previous study has found out that miR-144-3p was highly up-regulated in type 2 diabetes (T2D) and could impair insulin signaling (Karolina et al., 2011). The phenotype of insulin resistance was closely related to adipocyte differentiation and obesity (Kahn and Flier, 2000; Fu et al., 2005). Besides, the expression of miR-144-3p was positively correlated with adipocyte volume in both lean and obese pigs according to our previous study (Li et al., 2012). However, the epigenetic mechanism underlying the function of miRNA-144-3p in governing adipogenesis is not well clarified at present. Thus, *in vivo* and *in vitro* experiments were operated to explore the role of miRNA-144-3p in adipogenesis in this study. Our results indicate that miR-144-3p is an important positive regulator of adipogenesis. Luciferase reporter assays demonstrate Kruppel like factor 3 (Klf3) and carboxy-terminal binding protein 2 (CtBP2), the corepressors of C/EBPα (Sue et al., 2008; Wang et al., 2015), are direct target genes of miR-144-3p. Therefore, these results suggest that miR-144-3p may be a potential target for therapeutic intervention in obesity and metabolic syndrome.

## Materials and Methods

### Experiment Animals

All animal experimental procedures and sample collections were performed in accordance with the guidelines of Institutional Animal Care and Use Committee of College of Animal Science and Technology of Sichuan Agricultural University, Sichuan, China, under permit NO. DKY-B20131403 (Ministry of Science and Technology, China, revised in June 2004). In the obesity model study, two groups of 7-week-old male Kunming mice (*n* = 8) were fed with a high-fat diet (HFD) or received normal chow (NCW), respectively, for 3 months. In the *in vivo* assay, two groups of male Kunming mice (*n* = 3) were tail-vein injected with miR-144-3p agomir or agomir control (RiboBio, Guangzhou, China), respectively. Injections were given every 3 days and lasted for 3 weeks, with a dose of 80 mg/kg body weight. During the experiment, mice were given free access to food and water under controlled light and temperature conditions. Mice were sacrificed by cervical dislocation, and adipose samples were collected for RNA extraction and histological analysis.

### Cell Culture and Transfection

3T3-L1 cells were maintained, differentiated, and transfected as described in our previous study (Shen et al., 2018). Briefly, 3T3-L1 cells were maintained in DMEM containing 100 U/ml penicillin, 100 μg/ml streptomycin, and 10% fetal bovine serum at 5% CO_2_ humidified atmosphere (37°C). For differentiation, cells were cultured in DMEM supplemented with 10% fetal bovine serum and MDI (1 μM dexamethasone, 0.5 mM 3-isobutyl-1-methylxanthine, 1 μM dexamethasone, and 5 μg/ml insulin) when cells reached confluence. After 2 days, the culture medium was replaced with DMEM containing 10% FBS and 5 μg/ml insulin every 48 h until the pre-adipocytes totally differentiated into mature adipocytes (day 8). For transfection, short double-stranded RNAs (miRNA mimics) and their OMe-modified antisense oligonucleotides (miRNA inhibitors) of miR-144-3p were synthesized by Ribobio (Guangzhou, China). The first transfection was operated when 3T3-L1 reached confluence (begin to differentiate). The transfection was carried out using lipid carrier lipofectamine 2000 (Invitrogen, Carlsbad, CA, United States) following the manufacturer’s instructions. Briefly, mimics or inhibitors (20 nM) were mixed with lipid carrier (2:1, v/v), then incubated in Opti-MEM (Invitrogen, Carlsbad, CA, United States) medium for 20 min. Then, 3T3-L1 cells were incubated with the oligonucleotides and lipofectamine complex for 5 h transfection course, and then replaced the mixtures with normal differentiation medium. The transfection was carried out every second day when the medium was replaced.

### Oil Red O Staining and Triglyceride Assay

Briefly, differentiated 3T3-L1 cells were gently washed twice with cold 1 × PBS, fixed in 4% paraformaldehyde for 30 min, and washed twice with deionized water. Then, cells were stained with 60% saturated Oil Red O for 1 h, washed twice with deionized water, and photographed using an Olympus IX53 microscope (Olympus, Tokyo, Japan). For the triglyceride assay, cells were washed with cold 1 × PBS, detached from the plates using a cell scraper, and homogenized by sonication (1 min). Total triglyceride quantification was performed with Infinity Triglycerides Reagent (NO. TR22421, ThermoFisher Scientific, San Jose, CA, United States) according to the manufacturer’s protocol.

### CCK-8 and EdU Assays

The proliferation rates of 3T3-L1 were measured using Cell Counting Kit-8 (CCK-8; Dojindo, Tokyo, Japan) according to the manufacturer’s instructions. Briefly, transfected cells (miR-144-3p mimic and inhibitor) were seeded in 96-well plates with a density of 2000 cells/well, then incubated in the growth medium containing 10% CCK-8. Cell samples were incubated at 37°C and collected at 6, 12, 24, 48, and 96 h. The absorbance of the sample was measured at 450 nm using a microplate reader (Bio-Tek Instruments, Inc., Winooski, VT, United States). For Edu assay, transfected cells (24 h post-transfection) were incubated in the growth medium containing 50 mM Edu reagent (RiboBio, Guangzhou, China). After 24 h Edu staining, samples images were captured using a Nikon TE2000 microscope (Nikon, Tokyo, Japan).

**Table 1:** The primer sequences used for q-PCR.

Gene	Primer Sequence (5′-3′)	TM/°C
PPAR-γ	F: CGCTGATGCACTGCCTATGA	59
	R: AGAGGTCCACAGAGCTGATTCC	
C/EBP-α	F: CGCAAGAGCCGAGATAAAGC	59
	R: CACGGCTCAGCTGTTCCA	
aP2	F: CGATCCCAATGAGCAAGTGG	63
	R: TGGGTCAAGCAACTCTGGAT	
Klf3	F: CGGTGGACCTCACGGTGAACAA	63
	R: GCCATCACAGGCGGCATAGACA	
CtBP2	F: CTTTGGATTCAGCGTCAT	58
	R: GGTTGTGCTCGTTCAGG	
CycD1	F: TAGGCCCTCAGCCTCACTC	63
	R: CCACCCCTGGGATAAAGCAC	
CycE	F: CAGAGCAGCGAGCAGGAGC	60
	R: GCAGCTGCTTCCACACCACT	
CDK4	F: GTCAGTTTCTAAGCGGCCTG	62
	R: CACGGGTGTTGCGTATGTAG	
ACS	F: CGCACCCTTCCAACCAACA	62
	R: CGCTATTTCCACTGACTGCAT	
ELOVL6	F: AAGCACGCTCTATCTCCTGTT	60
	R: CTGCGTTGTATGATCCCATGAA	
SCD-1	F: TTCTTGCGATACACTCTGGTGC	62
	R: CGGGATTGAATGTTCTTGTCGT	
ACOX2	F: AAACACTGAGCTGCGGAGAA	60
	R: AATGCGTTCAGGACCGTCTT	
ACADL	F: ACATACAGACGGTGCAGCAT	60
	R: TCCGTGGAGTTGCACACATT	
miR-144-3p	F: TACAGTATAGATGATGTACT	60
	R: Uni-miR qPCR Primer, included in the kit	
β-actin	F: TGGAATCCTGTGGCATCCATGAAAC	60
	R: TAAAACGCAGCTCAG TAACAGTCCG	
U6	F: CTCGCTTCGGCAGCACA	60
	R: AACGCTTCACGAATTTGCGT	
WT- Klf3^∗^	F:CCGCTCGAGCGGTAAGACTGAATGGGTAAAGG	60
	R: ATATGCGGCCGCTATAACTATGACATATATCATATAC	
WT- CtBP2^∗^	F:CCGCTCGAGCGGGACGAGAAGGGTTCAAGAAG	60
	R: ATATGCGGCCGCTATAATACAAAGGCTCAAAATAGGC	

*Marked by an asterisk are the primers for amplifying 3′-UTR of SP1 and CtBP2 (F, forward; R, reverse). Gene abbreviation: PPAR-γ, peroxisome proliferator-activated receptor-g coactivator; C/EBP-α, CCAAT/enhancer-binding proteins alpha; aP2, adipocyte fatty-acid binding protein 2; Klf3, Kruppel-like factor 3; CtBP2, C-terminal binding protein 2; CycD1, Cyclin D1; CycE, Cyclin E; CDK4, cyclin-dependent kinase 4; ACS, acetyl-CoA synthetase; ELOVL6, ELOVL fatty acid elongase 6; SCD-1, stearoyl-Coenzyme A desaturase 1; ACOX2, acyl-CoA oxidase 2;ACADL, acyl-Coenzyme A dehydrogenase, long-chain; β-actin, actin beta.*

**FIGURE 1:**
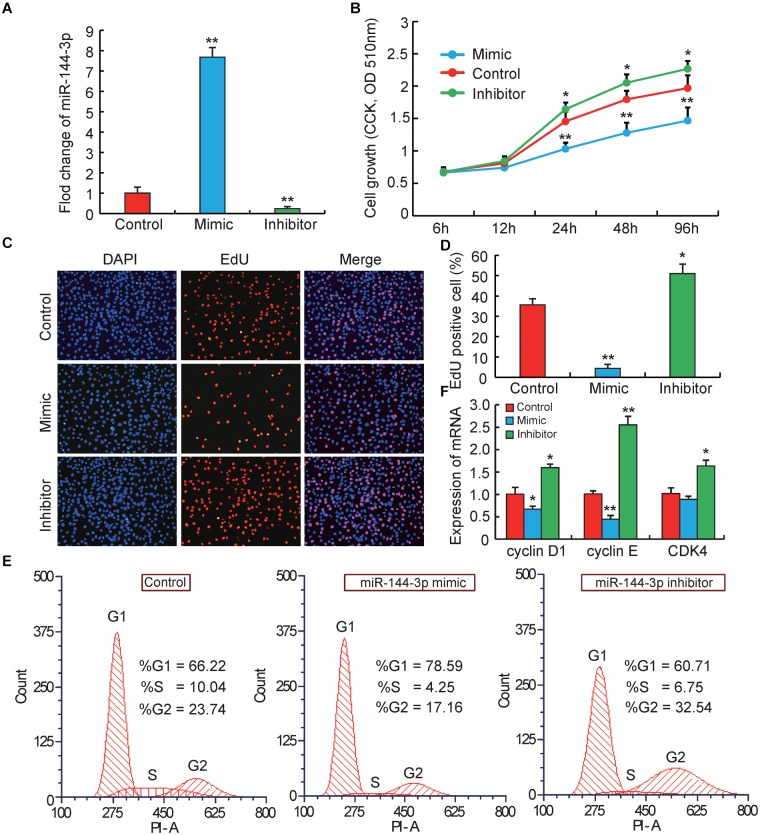
miRNA-144-3p inhibits pre-adipocyte proliferation. **(A)** The transfection efficiency of 3T3-L1 cells transfected with miR-144-3p mimic or inhibitor. **(B)** Cell proliferation was evaluated by CCK8. **(C,D)** Cell proliferation was evaluated by EdU staining. **(E)** The cell cycle phases of 3T3-L1 cells transfected with miR-144-3p mimic, inhibitor and negative control, analyzed by flow cytometry. **(F)** The relative expression of cell cycle regulatory genes (Cyclin D1, Cyclin E, and CDK4). All data were expressed as means ± SD (error bars represent the SD from three independent experiments).^∗^*p* < 0.05, ^∗∗^*p* < 0.01.

### Quantitative RT-PCR Analysis

Quantitative RT-PCR (q-PCR) was used to measure mRNA and microRNA (miRNA) expression. Total RNA was extracted from adipose tissue and cell samples using Trizol Reagent (Invitrogen, Carlsbad, CA, United States), and was further purified with RNeasy columns (Qiagen, CA, United States) according to the manufacturer’s protocol. Subsequently, mRNA and miRNA were reverse-transcribed to cDNA using the PrimeScript^TM^ RT Master Mix Kit and the PrimeScript miRNA RT-PCR Kit, respectively (TaKaRa, Dalian, China). Quantitative PCR was performed using the SYBR Green Real-time PCR Master Mix (TaKaRa, Dalian, China) by CF96 Real-Time PCR Detection System (Bio-Rad, Hercules, California, United States). β-actin and U6 were served as endogenous controls for mRNA and miRNA expression, respectively. The 2^−ΔΔCt^ method was used to calculate the relative expression levels of mRNA and miRNA (Livak and Schmittgen, 2001). All PCR primer sequences were shown in Table 1.

### Luciferase Reporter Assay

The wild-type 3′UTR of Klf3 (WT-Klf3) and CtBP2 (WT-CtBP2) were amplified by RT-PCR from genomic DNA of 3T3-L1 cells. The RT-PCR products were inserted between XhoI and NotI restriction sites of psiCHECK^TM^-2 vector (Promega, Madison, WI, United States), and validated by sequencing. Mutated plasmids were mutated at positions 1-7 of the seed match using a commercial mutagenesis kit (TransGen Biotech, Beijing, China), termed mutant-type Klf3 (Mut-Klf3) and CtBP2 (Mut-CtBP2), and also validated by sequencing. For the luciferase reporter analysis, 2 × 10^4^ HeLa cells were plated in a 96-well plate. One day after seeding, HeLa cells were, respectively, co-transfected with WT-Klf3 (WT-CtBP2 or Mut-Klf3 or Mut-CtBP2) and miR-144-3p mimic. Cells were harvested 48 h after transfection and subjected to Renilla and firefly luciferase activity using the Dual-Luciferase Reporter Assay System (Promega, United States) following the manufacturer’s instructions. All transfection and luciferase analysis experiments were conducted in triplicate.

### Cell Cycle Assay

Briefly, cell samples were fixed in chilled ethanol overnight after resuspending in PBS with 1% normal goat serum (NGS). After two rounds of washing, cells were resuspended in PBS with 1% NGS with 120 μg ml^−1^ propidium iodide and 10 μg ml^−1^ RNase A, and then incubated for 30 min at 37°C. Subsequently, the cell cycle was measured by flow cytometry (FACScan, BD Biosciences).

### Western Blotting

Briefly, cells were lysed on ice using a commercial lysis buffer (Sigma, Louis, Mo, United States), according to the manufacturer’s instructions. Collected proteins were boiled in 5× SDS buffer for 5 min, then separated by 10% SDS-polyacrylamide gel, and transferred to a PVDF membrane (Bio-Rad Co., United States). Subsequently, transferred membranes were blocked by TBST containing 5% non-fat dried milk for 2 h at 37°C, and then immunoblot (incubated overnight at 4°C) was conducted with primary antibody (Abcam, Shanghai, China). Then, immunoblot membranes were washed three times with TBST for 15 min and then incubated with horseradish peroxidase-conjugated secondary antibody for 1 h at 37°C. The blots were visualized by DAB reagent (Boster, Wuhan, China) according to the manufacturer’s instructions.

### Statistical Analysis

Data were analyzed by SPSS software (21.0 version). All data were presented as means ± standard deviation (*SD*). Differences in groups were analyzed with Student’s *t*-test. Differences were considered statistically significant at *p* < 0.05.

**FIGURE 2:**
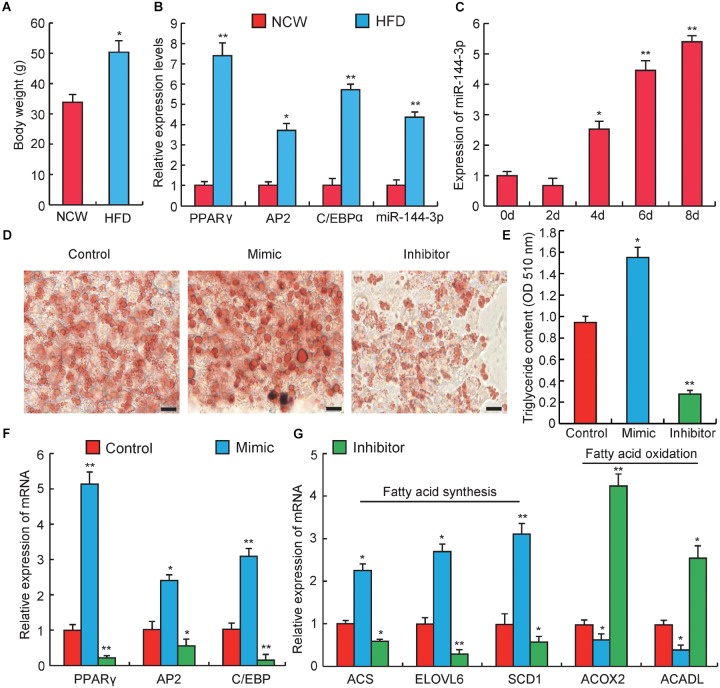
miRNA-144-3p promotes adipocyte differentiation. **(A)** The body weight of Kunming mice after feeding 3 months of high-fat diets (HFD) or normal chow (NCW). **(B)** Adipose tissue expression of adipogenic marker genes (PPARγ, C/EBPα, aP2) and miR-144-3p in HFD and NCW fed mice. **(C)** The relative expression of miR-144-3p during pre-adipocyte differentiation. **(D)** Oil Red O staining of terminally differentiated adipocytes (Day 8). **(E)** The contents of triglycerides in terminally differentiated adipocytes. **(F)** The relative mRNA expression levels of adipogenic marker genes PPARγ, AP2, and C/EBPα in 3T3-L1 transfected with miR-144-3p mimic or inhibitor. **(G)** The expression levels of genes related to fatty acid oxidation and fatty acid synthesis in terminally differentiated cells (Day 8) transfected with miR-144-3p mimic, inhibitor, and negative control. Scale bar, 10 μm. All data were expressed as means ± SD (error bars represent the SD from three independent experiments). ^∗^*p* < 0.05, ^∗∗^*p* < 0.01.

## Results and Discussion

### miR-144-3p Inhibits 3T3-L1 Pre-adipocyte Proliferation

It is well known that the biological process of adipocyte proliferation and differentiation is the foundation for the accumulation of lipids in adipose tissue (Rosen and MacDougald, 2006). However, miRNAs that related to regulating pre-adipocyte proliferation and differentiation were proved to have opposite effects. For example, miR-125b-5p and miR-26b could inhibit pre-adipocytes proliferation but promote the differentiation (Song et al., 2014; Ouyang et al., 2015). miR-199 and miR-125a-5p could promote pre-adipocytes proliferation but inhibit the differentiation (Shi et al., 2014; Xu et al., 2018). In this study, to explore the potential role of miR-144-3p in the proliferation of pre-adipocytes, firstly 3T3-L1 pre-adipocytes were used to transiently transfect miR-144-3p mimic or inhibitor, respectively. 3T3-L1 pre-adipocyte cell line is a widely used adipocyte model, which is an ideal approach to understand the molecular mechanism governing adipogenesis (Farmer, 2006). The transfection efficiency in 3T3-L1 was shown in Figure 1A, miR-144-3p mimics or inhibitors significantly increased (approximate seven times) or suppressed (approximate nine times) the expression levels of miR-144-3p when compared to the negative control group, respectively. These data suggested that the transfection experiment operated in this study was a great success and ensured the data reliability in subsequent experiments. Next, Counting Kit 8 (CCK-8) and 5-ethynyl-20-deoxyuridine (EdU) staining were also used to evaluate the function of miR-144-3p on pre-adipocyte proliferation. As shown in Figure 1B, after 24 h transfection, the growth rate of 3T3-L1 pre-adipocytes was significantly decreased or increased in mimics or inhibitor group, respectively, when compared to the control group. This finding was also confirmed by EdU analysis. As shown in Figures 1C,D, overexpression of miR-144-3p could significantly suppress the number of EdU-positive cells when compared to the control group. However, knockdown of miR-144-3p significantly increased the ratio of EdU-positive cells. Besides, to confirm the function of miR-144-3p on pre-adipocyte proliferation, the expression levels of some important cell cycle regulatory factors were also detected. For example, cyclin-dependent kinases (such as CDK4), Cyclin D1 and Cyclin E have been recognized as key regulators of cell growth and proliferation in eukaryotes, which are required for G1/S and G2/M transitions in mammalian cells (Resnitzky et al., 1994; Suzuki et al., 2000). As shown in Figure 1F, the results are consistent with the observations above, and qRT-PCR analysis indicated that knockdown of miR-144-3p could remarkably increase the Cyclin D1, Cyclin E, and CDK4 expression. While overexpression miR-144-3p significantly suppressed the expression of these cell cycle regulatory factors. Furthermore, the cell cycle distribution was investigated with miR-144-3p overexpression or knockdown, respectively. Flow cytometry analysis showed that overexpression of miR-144-3p could increase the ratio of cells in the G0/G1 phase and decrease the ratio of cells in the G2/M phases, and vice versa in the miR-144-3p knockdown group (Figure 1E). Therefore, these results collectively suggest that miR-144-3p may inhibit 3T3-L1 pre-adipocyte proliferation.

**FIGURE 3:**
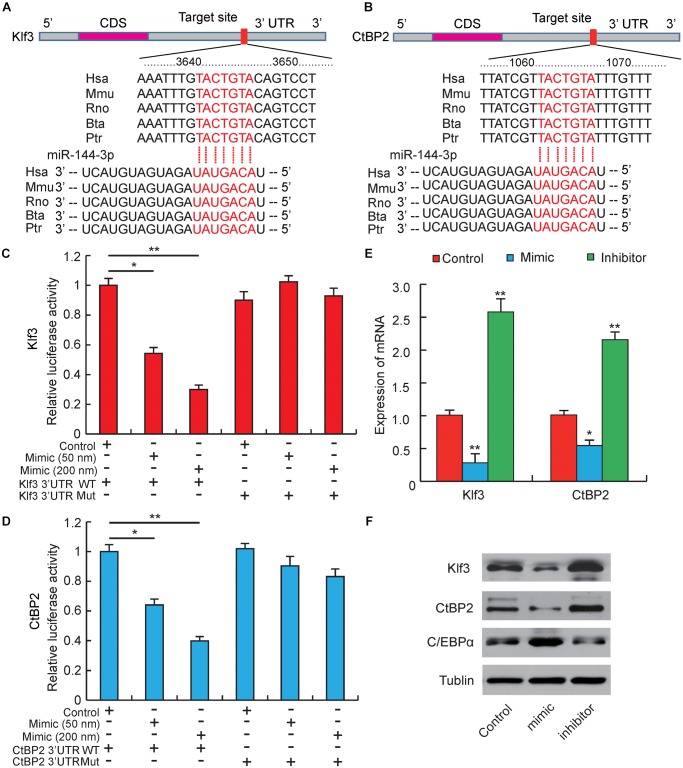
miR-144-3p targets the 3′-UTR of Klf3 and CtBP2. **(A)** Sequence alignments of miR-144-3p with 3′-UTR of human (Hsa), mouse (Mmu), rat (Rno), cattle (Bta), and chimpanzee (Ptr) Klf3 mRNA. **(B)** Sequence alignments of miR-144-3p with 3′-UTR of human (Hsa), mouse (Mmu), rat (Rno), cattle (Bta), and chimpanzee (Ptr) CtBP2 mRNA. Binding site and seed region of miR-144-3p are indicated in red. **(C)** The repressive effect of miR-144-3p on the activity of Klf3 3′UTR measured by luciferase assay. **(D)** The repressive effect of miR-144-3p on the activity of CtBP2 3′UTR measured by luciferase assay. **(E)** The influence of miR-144-3p mimic and inhibitor on Klf3 and CtBP2 mRNA expression. **(F)** Western blot analysis on the expression of Klf3, CtBP2, and C/EBPα in 3T3-L1 cells treated with miR-144-3p mimic or inhibitor. All data were expressed as means ± SD (error bars represent the SD from three independent experiments).^∗^*p* < 0.05, ^∗∗^*p* < 0.01.

**FIGURE 4:**
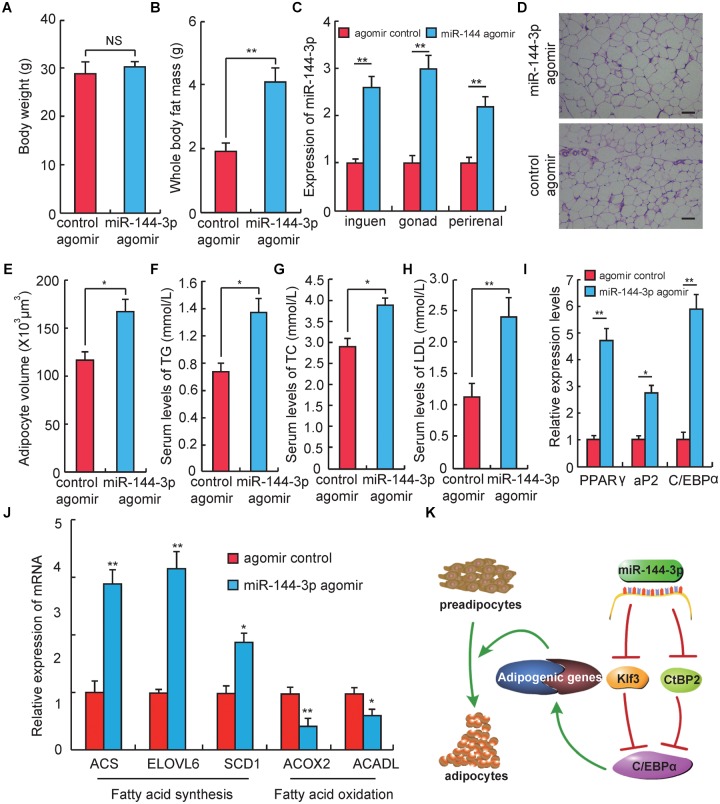
miR-144-3p promotes adipogenesis *in vivo*. **(A)** The body weight of Kunming mice after 3 weeks of tail vein injecting miR-144-3p agomir and negative control. **(B)** Whole body fat mass of Kunming mice after 3 weeks of tail-vein injecting with miR-144-3p agomir and negative control. **(C)** The expression levels of miR-144-3p in different adipose tissues from mice injected with miR-144-3p agomir and negative control. **(D)** HE staining of gonads fat tissue from mice injected with miR-144-3p agomir and negative control. Scale bar, 50 μm. **(E)** The average adipocyte volume of gonads fat from mice injected with miR-144-3p agomir and negative control. **(F–H)** The serum levels of total triglyceride (TG), cholesterol (TC), and low-lipoprotein (LDL) in mice injected with miR-144-3p agomir and nagative control. **(I)** Adipose tissue expression of adipogenic marker genes (PPARγ, C/EBPα, aP2) in miR-144-3p agomir and NC mice. **(J)** The expression levels of genes related to fatty acid oxidation and fatty acid synthesis in gonads fat tissue in mice injected with miR-144-3p agomir and negative control. **(K)**. One of the pathway of miR-144-3p to promote adipocyte differentiation. All data were expressed as means ± SD (error bars represent the SD from three independent experiments). ^∗^*p* < 0.05, ^∗∗^*p* < 0.01.

### miR-144-3p Promotes 3T3-L1 Pre-adipocyte Differentiation

To further investigate the effect of miR-144-3p on adipogenesis, obese mice were induced by a high-fat diet (HFD). As shown in Figure 2A, the body weight significantly increased in HFD feeding group when compared to the normal chow (NCW) receiving group (*p* < 0.05), which indicated that mice could become obese under HFD. As expected, the expression levels of two key adipogenic genes, PPARγ and C/EBPα (Farmer, 2006), and an adipocyte-specific gene, aP2 (Hotamisligil et al., 1996), were significantly elevated in the HFD group when compared to the NCW group. Interestingly, the expression level of miR-144-3p significantly increased in the adipose tissue from HFD-fed mice (Figure 2B). This finding also agreed with our previous study, which reported that miR-144-3p expression had a positive correlation with adipocyte volume in both lean and obese pigs (Li et al., 2012). Subsequently, to test whether the expression pattern of miR-144-3p *in vivo* could also be observed *in vitro*, the expression of miR-144-3p was investigated during 3T3-L1 pre-adipocyte differentiation. As shown in Figure 2C, the expression level of miR-144-3p markedly increased during adipogenic differentiation. As expected, overexpression of miR-144-3p could significantly promote lipid accumulation in 3T3-L1 and accelerate the process of adipogenesis according to the Oil Red O staining analysis (Figure 2D). In accordance with these findings, the triglyceride content in 3T3-L1 cells was also significantly increased in the miR-144-3p mimic group (*p* < 0.05), and significantly decreased in the inhibitor group (*p* < 0.01) (Figure 2E). To further confirm the function of miR-144-3p on adipogenesis, expression levels of some adipogenesis related regulators and markers were detected. As shown in Figure 2F, the expression of aP2, C/EBPα, and PPARγ had higher levels in the miR-144-3p mimic group when compared to the control group, and an opposite result was observed in inhibitor group. Moreover, fatty acids served as an important component in adipose tissue, and both increasing fatty acid synthesis and decreasing fatty acid oxidation can enhance lipid accumulation in adipocytes (Hems et al., 1975; Abu-Elheiga et al., 2001). Here, as expected, the expression of genes involved in fatty acid synthesis (Du et al., 2018) was significantly increased in the mimic group, but the expression of genes associated with fatty acid oxidation (Xu et al., 2018) was suppressed in the mimic group (Figure 2G). Taken together, these results indicate that miR-144-3p can promote the differentiation of 3T3-L1 pre-adipocytes by diminishing fatty acid production and enhancing fatty acid oxidation.

### miR-144-3p Promotes 3T3-L1 Adipogenesis by Targeting Klf3 and CtBP2

Our studies above have proved that miR-144-3p could inhibit pre-adipocytes proliferation and promote its differentiation, but the target genes regulated by miR-144-3p were still unclear (Ling et al., 2011). Therefore, to further explore the potential epigenetic mechanism underlying miR-144-3p promoting 3T3-L1 adipogenesis, computational prediction programs, like Pic Tar, targetScan, and miRanda, were used to predicate potential target genes of miR-144-3p. All the target genes must be perfectly matched to the seed sequence (2–8 site) of miR-144-3p by each software. As shown in Figures 3A,B, among the predicted target genes, both Klf3 and CtBP2 contained a complementary seed sequence of miR-144-3p in the 3′-UTR region. Interestingly, a previous study reported that Klf3 could directly bind to the endogenous C/EBPα promoter and repress its activity, and overexpression of Klf3 could suppress adipogenesis in 3T3-L1 cells (Sue et al., 2008). Zhu et al. (2018) also proved that miR-20a could promote adipogenic differentiation by targeting Klf3 in bone marrow stromal cells. CtBP family proteins are known as transcriptional corepressors (Chinnadurai, 2002; Vernochet et al., 2009). Previous studies have found out that CtBP2 could directly bind to the adipogenic maker (C/EBPα) and down-regulate its expression, which results in suppressing adipocyte differentiation (Wang et al., 2015). Interestingly, a previous study reported that Klf3 repressed transcription accompanying by recruiting CtBP corepressors in adipogenesis (Sue et al., 2008). In this study, homology analysis was used, and it proved that the two complementary sites between Klf3/CtBP2 and miR-144-3p were highly conserved among different species (Figures 3A,B). This result suggests that the epigenetic regulation is possibly a universal model in mammals. Furthermore, a double fluorescent reporter assay was performed to confirm that miR-144-3p suppressed Klf3 and CtBP2 activity by binding to the 3′-UTR. As shown in Figures 3C,D, the relative luciferase activity was significantly decreased in HeLa cells co-transfected with WT-Klf3/WT-CtBP2 and miR-144-3p mimic (*p* < 0.05), and the reducing of luciferase activity had a dose-dependent manner of mimic concentration. However, the luciferase activity didn’t decrease in HeLa cells co-transfected with MUT luciferase plasmids (Figures 3C,D). Furthermore, overexpression or knockdown of miR-144-3p in 3T3-L1 cells could significantly suppress or promote Klf3/CtBP2 expression in mRNA level and protein level, respectively (*p* < 0.01) (Figures 3E,F). Besides, considering both Klf3 and CtBP2 are corepressors of C/EBPα, C/EBPα expression was detected in 3T3-L1 transfected with miR-144-3p mimic and inhibitor. In agreement with the conjecture, the protein expression level of C/EBPα, a crucial and pivotal regulator of adipogenesis (MacDougald et al., 1995), was significantly promoted by miR-144-3p (Figure 3F). Taken together, these data suggest that miR-144-3p promotes adipocyte differentiation by directly targeting the 3′-UTR of Klf3 and CtBP2, which results in releasing C/EBPα from Klf3 and CtBP2.

### miR-144-3p Promotes Adipogenesis *in vivo*

To test whether the effect of miR-144-3p on adipogenesis in cell culture could also be observed *in vivo*, miR-144-3p expression *in vivo* was up-regulated by injecting miR-144-3p agomir in mice through the tail vein. Three weeks after the first injection (corresponding to 3 days after the last injection), the mouse body weight had no significant difference between the miR-144-3p agomir and negative control group. But the whole body fat mass in the miR-144-3p agomir group was significantly higher than the negative control (Figures 4A,B). To confirm that miR-144-3p was successfully overexpressed in adipose tissue by tail injection of agomirs, the expression levels of miR-144-3p in multiple adipose tissues were studied. As shown in Figure 4C, the expression level of miR-144-3p significantly heightened in inguen, gonad, and perirenal fat when mice were tail-injected with miR-144-3p agomirs (*p* < 0.01). Based on this finding, the adipocyte volume of gonads fat tissue between two different groups was also measured. As shown in Figures 4D,E, mice injected with miR-144-3p agomir have a higher adipocyte volume than that in the negative control mice. Besides, miR-144-3p agomir could significantly increase serum levels of total TC, TG, and LDL when compared to the negative control (Figures 4F–H). These blood indicators were demonstrated to be related to the phenotype of obesity in previous studies (Tan et al., 2017; Du et al., 2018). Therefore, these studies suggest that miR-144-3p also have the function of promoting adipogenesis *in vivo*. To further confirm this finding, the expression levels of genes related to adipogenesis of the two groups were also measured. As shown in Figure 4I, the expression levels of three key adipogenic genes (PPARγ, C/EBPα, and aP2) were elevated in the agomir group when compared to the negative control group. For example, PPARγ is widely known as a necessary factor for both adipogenesis and HFD induced obesity (Kubota et al., 1999; Jones et al., 2005). Besides, PPARγ is involved in glucose metabolism via an improvement of insulin sensitivity (Kubota et al., 1999; He et al., 2003; Hevener et al., 2003). Therefore, synthetic PPARγ agonists (thiazolidinediones, glitazones) are clinically used as insulin sensitizers to treat patients with type 2 diabetes (Kahn et al., 2006). Furthermore, miR-144-3p also significantly promoted the expression of genes associated with fatty acid synthesis and repressed the expression of genes involved in the fatty acid oxidation, when compared to the control group (Figure 4J). As expected, the research confirmed that the effect of miR-144-3p on adipogenesis in 3T3-L1 could also be observed *in vivo*. Taken together, these data elucidate a possible pathway used by miR-144-3p to regulate adipocyte differentiation, and the plausible regulatory network is shown in Figure 4K.

## Conclusion

In summary, our results confirmed that miR-144-3p inhibited 3T3-L1 pre-adipocyte proliferation, and promoted differentiation by directly targeting Klf3 and CtBP2. miR-144-3p would promote C/EBPα expression by releasing Klf3 and CtBP2 from C/EBPα promoter region. Furthermore, miR-144-3p was shown to up-regulate genes associated with fatty acid synthesis, and to down-regulate genes involved in fatty acid oxidation. In addition, the effect of miR-144-3p on adipogenesis can also be observed *in vivo* by injecting with miR-144-3p agomir through the tail vein. Thus, deeper studies regarding the *in vivo* function of miR-144-3p should be performed to evaluate this molecule as a potential target for obesity.

## Ethics Statement

This study was carried out in accordance with the recommendations of ‘Institutional Animal Care and Use Committee of College of Animal Science and Technology of Sichuan Agricultural University (NO. DKY-B20131403), Ministry of Science and Technology’. The protocol was approved by the ‘Ministry of Science and Technology’.

## Author Contributions

LS and LZ conceived and designed the experiments. LS, QL, JW, YZ, and LN performed the experiments. LS, QL, LB, SS, and JW analyzed the data. XL and SZ contributed reagents, materials, and analysis tools. LS and SZ wrote the paper.

## Conflict of Interest Statement

The authors declare that the research was conducted in the absence of any commercial or financial relationships that could be construed as a potential conflict of interest.
